# Improved neurocognitive functions correlate with reduced inflammatory burden in atrial fibrillation patients treated with intensive cholesterol lowering therapy

**DOI:** 10.1186/1742-2094-10-78

**Published:** 2013-06-28

**Authors:** Knut Tore Lappegård, Monica Pop-Purceleanu, Waander van Heerde, Joe Sexton, Indira Tendolkar, Gheorghe Pop

**Affiliations:** 1 Divison of Internal Medicine, Nordland Hospital, Norway and University of Tromsø, Bodø, Norway; 2 Department of Psychiatry, Radboud University Medical Center, Nijmegen, The Netherlands; 3 Department of Laboratory Medicine, Laboratory of Hematology, Radboud University Medical Center, Nijmegen, The Netherlands; 4 Department of Biostatistics, University of Oslo, Gaustad, Sognsvannsveien 9, 0372 Oslo, Norway; 5 Donders Institute for Brain, Cognition and Behaviour, Center for Cognitive Neuroimaging, Nijmegen, The Netherlands; 6 Department of Cardiology, Radboud University Medical Center, Nijmegen, The Netherlands

**Keywords:** Atrial Fibrillation, Inflammation, Neurocognitive Function, Cerebral MRI, Statins

## Abstract

**Background:**

Atrial fibrillation (AF) is associated with increased mortality and morbidity, including risk for cerebral macro- and microinfarctions and cognitive decline, even in the presence of adequate oral anticoagulation. AF is strongly related to increased inflammatory activity whereby anti-inflammatory agents can reduce the risk of new or recurrent AF. However, it is not known whether anti-inflammatory therapy can also modify the deterioration of neurocognitive function in older patients with AF. In the present study, older patients with AF were treated with intensive lipid-lowering therapy with atorvastatin 40 mg and ezetimibe 10 mg, or placebo. We examined the relationship between neurocognitive functions and inflammatory burden.

**Findings:**

Analysis of inflammatory markers revealed significant reductions in high sensitivity C-reactive protein (hs-CRP), fibroblast growth factor (FGF), granulocyte colony-stimulating factor (G-CSF), granulocyte-macrophage colony-stimulating factor (GM-CSF), interleukin-1 receptor antagonist (IL-1RA), interleukin (IL)-9, IL-13 and IL-17, and interferon-γ (IFNγ) in the treatment group compared to placebo. Reduction in plasma concentration of IL-1RA, IL-2, IL-9 and IL-12, and macrophage inflammatory protein-1β (MIP-1β) correlated significantly with improvement in the neurocognitive functions memory and speed. Loss of volume in amygdala and hippocampus, as determined by magnetic resonance imaging (MRI), was reduced in the treatment arm, statistically significant for left amygdala.

**Conclusions:**

Anti-inflammatory therapy through intensive lipid-lowering treatment with atorvastatin 40 mg and ezetimibe 10 mg can modify the deterioration of neurocognitive function, and the loss of volume in certain cerebral areas in older patients with AF.

**Trial registration Clinical Trials.gov:**

NCT00449410

## Background

Atrial fibrillation (AF) is a common arrhythmia, and even with optimal treatment it is associated with considerable morbidity and mortality. Current therapies include anticoagulation, rhythm and rate control, and in selected cases pulmonary vein isolation and ablation. There is increasing interest in inflammatory aspects of AF [1,2], beneficial effects of anti-inflammatory agents [3-6] and blocking of the renin-angiotensin-aldosterone system (RAAS) to prevent the arrhythmia [7,8]. The aspect of inflammation as part of the general aging process has also been addressed recently [9,10]. Oral anticoagulation significantly reduces the risk of thromboembolic complications in AF, but even with an international normalized ratio (INR) within the therapeutic range strokes do occur. Furthermore, even in the absence of overt strokes, patients with AF have an increased risk of cognitive decline and development of dementia [11-16].

AF patients have elevated plasma concentration of several inflammatory markers, and in patients without AF a possible relation between these markers and Alzheimer’s disease has been suggested [17]. There is increasing evidence linking inflammation to thrombosis [1,18,19]. A possible explanation for the development of dementia in AF patients may be thrombotic microinfarctions triggered by inflammatory processes and not necessarily microemboli. We have recently shown that patients with AF on adequate oral anticoagulation remain in a prothrombotic state, as indicated by the presence of endogenous thrombin potential [20]. This was reduced by intensive lipid-lowering therapy with a combination of 40mg atorvastatin and 10mg ezetimibe. The therapy was associated with lower levels of a number of inflammatory parameters [20], as well as a reduced decline in several neurocognitive functions in association with changes in limbic regions mediating these functions [21]. However, it remains unclear whether the obvious clinical benefit of change in inflammatory markers in AF also relates to a positive effect on neurocognitive functions. We therefore extend our previous findings and report on the relationship between the inflammatory changes and neuropsychological performance, as well as structural changes of amygdala, hippocampus and white matter lesions, which are mainly associated with the neurocognitive decline in AF.

### Patients, materials and methods

#### Study design

The patients, blood analyses, electrocardiograms and echocardiograms in this study have been described in detail elsewhere [20]. The study complied with the Declaration of Helsinki and was approved by the medical ethics committees of the Radboud University Nijmegen Medical Centre and the Canisius Wilhelmina Medical Centre, both in Nijmegen, the Netherlands. Patients were included in the study only after having given a written consent of participation.Briefly, 34 patients with AF and without indication for lipid-lowering therapy were randomized to receive either atorvastatin 40 mg and ezetimibe 10 mg, or double placebo for 1 year. Baseline characteristics of the patients are given in Table 1. Prothrombin INR and dosage of coumarin/warfarin were similar in both groups and were unchanged throughout the study. Blood samples were collected at inclusion, and after 1, 3, 6, 9 and 12 months. Cytokines, chemokines and selected growth factors were analysed using a multiplex cytokine assay (Bio-Plex Human Cytokine 27-Plex Panel, Bio-Rad Laboratories Inc, Hercules, CA, USA) as previously described [20]. Complement activation products were determined in enzyme immunoassays as described [22]. Plasminogen activator inhibitor (PAI) was analysed in a Chromolize immunoassay (Biopool, Umeå, Sweden). Plasmin peak height was analysed using the Nijmegen Hemostasis Assay as described earlier [20,23].

**Table 1:** Baseline characteristics of treatment group and placebo

	**Treatment (n=17)**	**Placebo (n=17)**
Gender (males: females)	13: 4	14: 3
Age (years) (SD)	74.5 (4.2)	73.5 (4.0)
Smoking (n/total)	1/17	1/17
Hypertension (n/total)	12/17	13/17
BMI (kg/m^2^) (SD)	25.6 (3.4)	26.8 (8.3)
Chronic AF (n/total)	11/17	9/17
Duration of AF (years)(SD)	13.5 (15.0)	14.0 (19.3)

Data as mean (SD). AF, atrial fibrillation; BMI, body mass index;n, number of patients; SD, standard deviation.

#### *Neurocognitive test*s

All participants underwent a face-to-face clinical interview by a neuropsychologist, followed by a comprehensive neuropsychological assessment. The clinical interview ruled out a depressive disorder. The assessment was performed at baseline and 1year after enrolment in the study. An overview of the tests is given in Table 2.

**Table 2:** Overview of clinical and neuropsychological assessment

	**Measure**	**Domain**
Clinical	Multilevel Assessment Instrument	Instrumental activities of daily living
	Mini Mental State Examination	Cognitive screening
Memory	Rey Auditory Verbal Learning Test	Verbal memory
	Rey-Osterrieth Complex Figure Test	Visual memory, constructive ability
	WAIS-III: digit span	Working memory, concentration
Language	WAIS-III: vocabulary	Language, verbal expression
	Verbal fluency	Language, executive functioning
Executive function/speed	Reaction time tasks (ERT, SRT, CRT)	Reaction time, interference
	WAIS-III: digit symbol coding	Speed of information processing
	Stroop Color-Word Test I to III	Speed of information processing, interference
	Trail Making Test A	Motor speed, visual scanning
Executive function/switching	Trail Making Test B	Switching, visual scanning, motor speed
	Stroop Color-Word Test IV	Switching, speed of information processing, interference

CRT, choice reaction time; ERT, elementary reaction time; SRT, simple reaction time; WAIS, Wechsler Adult Intelligence Scale.

### Neuroimaging

All participants underwent T1 weighted and fluid attenuated inversion recovery at 1.5T with magnetic resonance imaging (MRI) scanning (Siemens, Munich,Germany) as described elsewhere [21].

### Statistics

To evaluate whether changes in brain areas were associated with treatment, the 12-month value of each area was regressed on its baseline value and a treatment indicator. The *P* value associated with the treatment indicator provides a test of whether changes in area-volume are associated with treatment.

The Spearman correlation between changes in cognitive test scores or MRI findings and changes in inflammatory parameters was computed. Since there were 35 different inflammatory parameters and a long range of cognitive tests, a number of statistically significant differences just by chance would be expected. To circumvent this potential pitfall, a single *P* value for all the different biomarkers was computed for each cognitive test and each domain [24]. The combined *P* values were computed using Tippett’s combination method [25] and its distribution under the null hypothesis of no association computed using the permutation distribution.

## Results

### Brain volume during treatment

Volumetric analyses revealed that both amygdala and hippocampal volume decreased during the study period. This was true for both the placebo arm and for patients receiving active treatment; however, the reduction was consistently smaller in the treatment group, although statistically significant (*P*<0.02) only for the left amygdala area (Table 3). Results were unaffected by adjustment for total brain volume.

**Table 3:** Volumes of selected brain areas at baseline and end of study

	**Treatment (μl)**		**Placebo (μl)**		***P *value**
	**Baseline**	**1 year**	**Baseline**	**1 year**	
Left amygdala	2,051	1,952	1,797	1,552	0.019
Left hippocampal	2,866	2,761	2,790	2,747	0.56
Right amygdala	2,082	1,992	1,829	1,628	0.20
Right hippocampal	2,944	2,866	2,972	2,814	0.50

### Combined *P* values for individual cognitive tests and domains

By combining the *P* values obtained for each individual Spearman correlation, it was possible to compute a combined *P* value for each cognitive test and domain. For the domains speed and memory, the correlation between rate of decline and changes in inflammatory markers was highly significant with *P* values of 0.02 and 0.03, respectively (Table 4). For the domains language, switching and attention, there was no correlation. Furthermore, for the individual Stroop Color-Word Test IV and Trail Making Test A, the *P* values were <0.01 and 0.02, respectively. The clinical interviews excluded a depressive disorder, and the Montgomery-Asberg Depression Rating Scale was below seven in all participants at baseline and at the end of the study.

**Table 4:** **Combined *P *values for the correlation between inflammatory parameters, cognitive domains and tests**

Attention 0.92	Speed 0.02	Language 0.12	Switching 0.12	Memory 0.03
SDST 0.21	Stro I 0.93	Stro IV 0.00	TMTA 0.02	
TMTB 0.83	WTDR 0.08	ERT 0.47	NPOSOM 0.23	

ERT, elementary reaction time; NPOSOM, abbreviation of the Dutch term for the total number of impaired neuropsychological tests; SDST, symbol digit substitution test; Stro I,Stroop Color-Word Test I; Stro IV,Stroop Color-Word Test IV; TMTA, Trail Making Test A; TMTB, Trail Making Test B; WTDR,15 word test delayed recall.

### Combined *P* values for individual inflammatory parameters

In the same manner as for the cognitive test, we pooled the *P* values for each inflammatory parameter across all the various tests and domains. Changes in interleukin-1 receptor antagonist (IL-1RA), interleukin (IL)-2, IL-9 and IL-12, and macrophage inflammatory protein-1β (MIP-1β) significantly correlated with changes in cognitive function with *P* values of 0.01, 0.04, 0.05, 0.04 and 0.01, respectively (Table 5). For the other inflammatory markers, no statistically significant correlations were found.

**Table 5:** ***P *values for each inflammatory parameter, pooled across all cognitive tests**

C1-inh 0.22	C3bc 0.77	C4bc 0.79	Properdin 0.56	TCC 0.93	PPH0.29	ETP 0.19
Eotaxin0.52	FGF 0.17	G-CSF 0.47	GM-CSF 0.41	IFNγ 0.19	MIP-1α 0.37	MIP-1β 0.01
IL-1β 0.18	IL-1RA 0.01	IL-2 0.04	IL-4 0.25	IL-5 0.11	IL-6 0.55	IL-7 0.69
IL-8 0.45	IL-9 0.05	IL-10 0.38	IL-12 0.01	IL-13 0.10	IL-15 0.56	IL-17 0.36
MCP-1 0.67	PAI 0.63	PDGF 0.44	RANTES 0.47	TNF-α 0.15	VEGF 0.40	IP-10 0.49

C1-inh, C1-inhibitor; ETP, endogenous thrombin potential; FGF, fibroblast growth factor; G-CSF, granulocyte colony-stimulating factor; GM-CSF, granulocyte-macrophage colony-stimulating factor; IFNγ, interferon-γ; IL, interleukin; IL-1RA, interleukin-1 receptor antagonist; IL-1β, interleukin-1β, IP-10 (also known as C-X-C motif chemokine 10or CXCL10), interferon gamma-induced protein 10; MCP-1 (also known as CCL2), monocyte chemotactic protein-1; MIP-1α (also known as CCL3), macrophage inflammatory protein-1α; MIP-1β (also known as CCL4), macrophage inflammatory protein-1β; PAI, plasminogen activator inhibitor; PDGF, platelet-derived growth factor; PPH, plasmin peak height; RANTES (also known as CCL5), regulated on activation, normal T cell expressed and secreted; TCC, terminal complement complex; TNF-α, tumor necrosis factor-α; VEGF, vascular endothelial growth factor.

### Combined *P* values for changes in MRI and inflammatory parameters

We found no statistically significant correlations between changes in white matter volume, white matter lesion volume or number of white matter lesions and changes in inflammatory parameters. Similarly, no consistent statistically significant correlations could be demonstrated when examining inflammatory parameters and white matter changes in individual cerebral regions, or between inflammatory parameters, amygdala and hippocampus volume (data not shown).

## Discussion

Even if optimally treated, AF is associated with increased morbidity and mortality. Recent studies have also indicated a higher prevalence of dementia among AF patients compared to matched controls [13,26]. Increased plasma concentration of certain cytokines is associated with dementia [17] and AF patients are known to have elevated levels of several cytokines [27,28]. Previous studies have shown that treatment with anti-inflammatory effects can prevent postoperative AF [6,29-31] and prevent recurrence after external cardioversion [32,33], but it is not known whether such treatment can also affect the negative consequences of the arrhythmia on neurocognitive functions.

We have recently shown that intensive lipid-lowering treatment reduces high sensitivity C-reactive protein (hs-CRP) and a number of other peripheral markers of inflammation in patients with AF [20]. Although a previous study [34] indicated that a combination of simvastatin and ezetimibe is no better than simvastatin alone in affecting intima-media thickness in patients with familial hypercholesterolemia, each statin and lipid-lowering treatment must be evaluated independently. Furthermore, our group of patients consisted of older AF patients without significantly elevated cholesterol levels and the achieved lipid-lowering effect was higher in our study compared to the previous. In the JUPITER trial, use of rosuvastatin was associated with a reduction in vascular events in a primary prophylaxis setting in patients with elevated C-reactive protein (CRP) [35]. In the present study, we demonstrate for the first time that there is a statistically significant correlation between reduction in a series of inflammatory markers and delayed neurocognitive decline, especially memory and speed of information processing in older patients with AF. However, whether this correlation indicates a causal relationship isunclear. Furthermore, from our results it is only possible to determine the combined effect of atorvastatin and ezetimibe, and not the individual contribution of each drug. The relative role of the various inflammatory markers is not clear, nor is the reason why some markers correlated with neurocognitive decline and some did not. It may be a result of the relatively low number of participants (type II error) but as will be seen below, other investigators have also demonstrated correlation between cognitive function and some, but not all, of the inflammatory markers measured.

An inverse relationship between plasma concentration of IL-2 and the probability of successful cardioversion after the administration of amiodarone has been demonstrated [36]. In our study, changes in IL-2 correlated with the rate of decline in cognitive function. To the best of our knowledge, the association between AF, neurocognitive function and the inflammatory markers, IL-1RA, IL-9, IL-12 and MIP-1β, has not previously been studied. In a recent study in rats, Ribeiro demonstrated that induction of a pro-inflammatory state by administration of metylmalonic acid resulted in increased levels of interleukin-1β(IL-1ß) and tumor necrosis factor-α (TNF-α), both in blood and in the cerebral cortex of the animals, as well as a reduction in spatial orientation [37].

We were unable to show any correlation between inflammatory markers and changes in white matter lesions of the brain. Furthermore, there were no statistically significant correlations between changes in inflammatory mediators and changes in amygdala and hippocampus volume. However, our data indicate that in the treatment group there was a trend towards a decrease in white matter lesions, while the placebo group showed an increase. One possibility is that the smaller initial white matter lesion volume attenuated the treatment effect. Another is that statistical power was insufficient due to the relatively small group of participants. Furthermore, the follow-up time may have been too short to detect differences in progress. The reason why the decline in only some domains and cognitive tests correlated with changes in inflammatory parameters is unclear. One possibility is that there are different mechanisms for the decline across the various functions, probably time-dependent, of which only some are susceptible to the effects of anti-inflammatory therapy. Another possible explanation could be the relatively low number of patients in our study, with a risk for type II statistical errors. However, the effect of powerful lipid-lowering treatment on inflammatory and hemostatic parameters reported previously [20], and the correlation between such findings and changes in cognitive functions, and certain brain volumes presented in this report, support the notion that there is a link between inflammation, thrombosis and dementia in AF. Thus, larger, long-term studies are warranted to confirm and extend our findings.

## Conclusions

In this study, anti-inflammatory therapy through intensive lipid-lowering treatment with atorvastatin 40 mg and ezetimibe 10 mg apparently modified the deterioration of neurocognitive function and the loss of volume in certain cerebral areas in older patients with AF.

## Abbreviations

AF: atrial fibrillation; BMI: body mass index; C1-inh: C1-inhibitor; CRP: C-reactive protein; CRT: choice reaction time; ERT: elementary reaction time; ETP: endogenous thrombin potential; FGF: fibroblast growth factor; G-CSF: granulocyte colony-stimulating factor; GM-CSF: granulocyte-macrophage colony-stimulating factor; hs-CRP: high sensitivity C-reactive protein; IFNγ: interferon-γ; IL: interleukin; IL-1RA: interleukin-1 receptor antagonist; IL-1β: interleukin-1β; INR: international normalized ratio; IP-10: interferon gamma-induced protein 10; MCP-1: monocyte chemotactic protein-1; MIP-1: macrophage inflammatory protein-1; MIP-1α: macrophage inflammatory protein-1α; MIP-1β: macrophage inflammatory protein-1β; MRI: magnetic resonance imaging; NPOSOM: abbreviation of the Dutch term for the total number of impaired neuropsychological tests; PAI: plasminogen activator inhibitor; PDGF: platelet-derived growth factor; PPH: plasmin peak height; RAAS: renin-angiotensin-aldosterone system; RANTES: regulated on activation, normal T cell expressed and secreted; SD: standard deviation; SDST: symbol digit substitution test; SRT: simple reaction time; TCC: terminal complement complex; TNF-α: tumor necrosis factor-α; VEGF: vascular endothelial growth factor; WAIS: Wechsler Adult Intelligence Scale; WTDR: 15 word test delayed recall.

## Competing interests

The authors report no conflicts of interest.

## Authors’ contributions

KTL performed analysis of inflammatory markers and wrote the paper. MP-P supervised the preparation and analysis of the psychological tests. WvH was responsible for the analysis of the hemostatic factors. JS performed the statistical analyses. IT supervised the analysis of the MRI scans. GP designed and initiated the study. All authors read and approved the final manuscript.
